# Ultrasound-guided microwave ablation for metastatic breast cancer: a 3-year follow-up case report and review of the literature

**DOI:** 10.3389/fonc.2026.1692301

**Published:** 2026-05-01

**Authors:** Juan Li, Binru Xia, Xiaojia Zhang, Lu Wang, Tingting Li, Jie Zou, Man Lu

**Affiliations:** Ultrasound Medical Center,Sichuan Cancer Hospital Institute, University of Electronic Science and Technology of China, Chengdu, China

**Keywords:** metastatic breast cancer, microwave ablation, minimally invasive therapy, oligometastasis, ultrasound-guided intervention

## Abstract

This case describes the application of ultrasound-guided microwave ablation (MWA) in the management of a solitary breast metastatic lesion in a 59-year-old female with a history of lung adenocarcinoma. Over a 36-month follow-up period, imaging and clinical assessments demonstrated sustained local tumor control without recurrence or major complications, highlighting MWA as a viable minimally invasive therapeutic option for metastatic breast cancer patients.

## Introduction

Metastatic breast carcinoma (MBC), defined as secondary malignant lesions originating from non-mammary primary tumors, represents an exceptionally rare entity of all malignant breast neoplasms (1–3). The primary tumors can originate from a wide array of sites, with the most common being contralateral breast cancer. Other sources include hematologic malignancies, melanoma, lung cancer, ovarian cancer, soft tissue sarcomas, among others (1, 4–7). Notably, The treatment of MBC primarily revolves around systemic therapy targeting the primary tumor, with local control measures, such as surgical resection, being opted for metastatic breast lesions (7). A systematic review identified no published cases utilizing microwave ablation (MWA), a modality offering superior thermal efficiency and real-time monitoring advantages (7). Herein, we present the first documented case of ultrasound-guided MWA for lung adenocarcinoma-derived MBC, highlighting its technical feasibility and short-term oncologic outcomes.

## Case presentation

The patient was diagnosed with stage IV lung adenocarcinoma (cT4N3M1) with multiple bilateral pulmonary metastases in July, 2021. Following multidisciplinary team discussion, four cycles of chemotherapy with pemetrexed plus cisplatin were administered according to NCCN guidelines (from August 10, 2021 to November 22, 2021). In December, 2021, intensity-modulated radiation therapy (IMRT, total dose 60 Gy in 30 fractions) was delivered to the primary right lung lesion. During the third week of radiotherapy, the patient reported a painless, firm, poorly mobile mass in the left breast. In January, 2022, a comprehensive breast evaluation was performed. Ultrasound revealed a 35×12×20 mm irregular mixed-echo mass with ill-defined margins at the 4–5 o’clock position of the left breast, 20 mm from the nipple. The lesion exhibited rich blood flow (Adler grade III) and was classified as Breast Imaging Reporting and Data System(BI-RADS) 4B.Contrast-enhanced breast MRI(CE-MRI) demonstrated a non-mass enhancement (32×15×24 mm) in the lateral quadrant of the left breast, showing isointensity on T1-weighted imaging (T1WI), mild hyperintensity on T2-weighted imaging (T2WI), and regional rapid enhancement on dynamic contrast-enhanced sequences with a “fast-in-fast-out” time-intensity curve. Diffusion-weighted imaging (DWI) revealed hyperintensity with a significantly reduced apparent diffusion coefficient (ADC) value (minimum ADC: 0.78×10^-^³ mm²/s), categorized as BI-RADS 4C. On January 18, 2022, an ultrasound-guided core needle biopsy of the left breast lesion was performed. Pathology confirmed metastatic adenocarcinoma, and immunohistochemistry supported a pulmonary origin (TTF-1+, NapsinA+, GATA3-, ER-). Due to patient refusal of repeat surgery and preference for minimally invasive therapy, ultrasound-guided MWA was proposed.

### Procedure

Following multidisciplinary evaluation, the oncology team recommended palliative ablation of the mass to alleviate symptoms and improve quality of life. Written informed consent was obtained from the patient and her legal guardians after detailed discussion of procedural risks and benefits. This study complied with the ethical principles outlined in the Declaration of Helsinki (2013 revision) and was approved by the Institutional Review Board of Sichuan Cancer Hospital. All procedures adhered to institutional operational guidelines for interventional oncology therapies.

**Figure 1:**
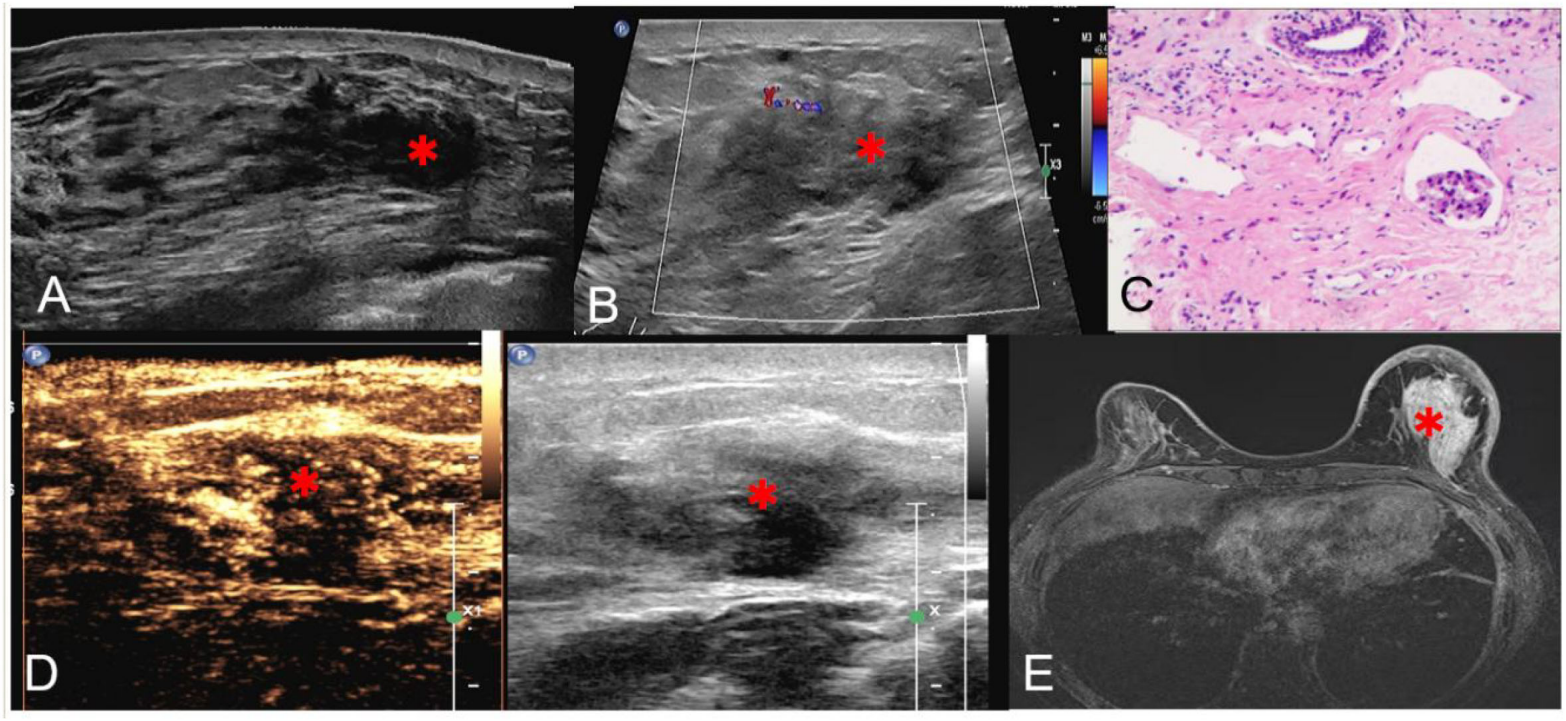
Pre-ablation imaging characteristics. **(A)** Panoramic imaging; **(B)** CDFI; **(C)** Pathological diagnosis; **(D)** Contrast-enhanced ultrasound(CEUS) demonstrating hypoenhanced lesion with extended enhancement; **(E)** MRI showing non-mass enhancement (*asterisk showed tumor area).

The ultrasound-guided ablation procedure was performed in February, 2022 by an interventional radiologist with over 10 years of experience in oncologic interventions. Preprocedural assessments, including complete blood count, coagulation profile, and electrocardiogram, confirmed the patient’s fitness for the intervention. Establish intravenous access preoperatively and prepare cardiac monitoring setup intraoperatively. The ablation trajectory was meticulously re-evaluated using conventional ultrasound and CEUS to optimize tumor targeting (**Figure 1**). Following aseptic preparation, A total of 20ml of mixed solution containing lidocaine, ropivacaine, and normal saline was injected respectively into the skin, retromammary space, and fascial space between the pectoralis major and minor muscles to perform local anesthesia and pectoral nerve block (Pecs block).

Under real-time ultrasound guidance, a 22-gauge introducer needle was advanced to the peritumoral region, through which a hydrodissection solution (saline-lidocaine mixture) was injected to establish a protective buffer zone isolating the tumor from adjacent normal tissues. Upon achieving adequate tissue separation, a microwave antenna was deployed into the lesion, and the moving-shot ablation technique was systematically applied along the tumor margins in a deep to superficial plane, simultaneously performing extended ablation of the tumor. MWA is terminated when vapor bubbles achieve complete coverage, followed by post-bubble dissipation contrast-enhanced ultrasound(CEUS) evaluation. Supplementary ablation is performed if incomplete treatment is identified, with final cessation contingent upon confirmed complete ablation on CEUS. Throughout the procedure and recovery period, patient maintained stable vital signs, and no procedural complications—including skin burns, hemorrhage, were observed.

### Assessment and follow-up

**Figure 2:**
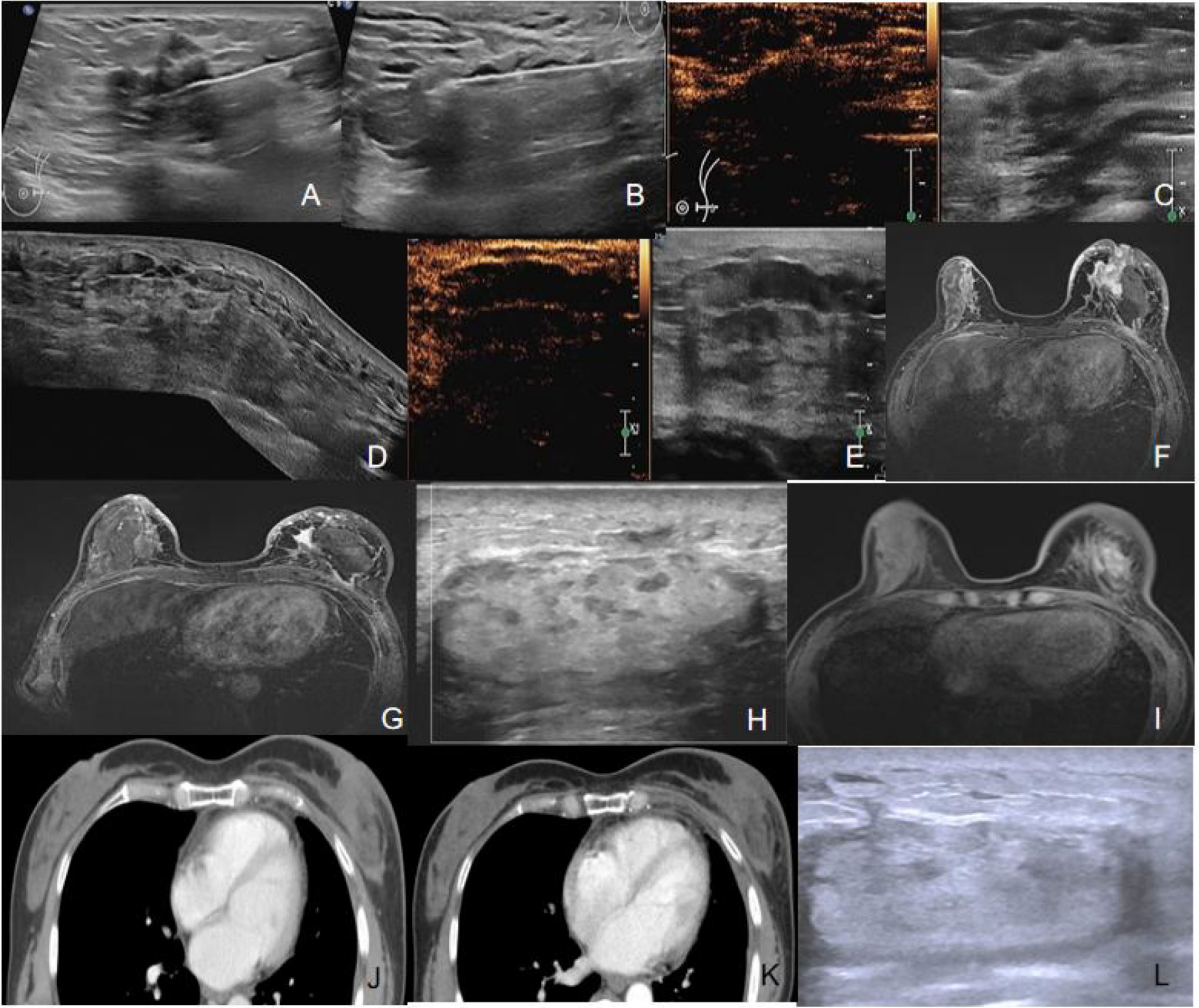
Long-term imaging follow-up after tumor ablation. **(A)** Pre-ablation biopsy; **(B)** Intraoperative ablation under ultrasound guidance; **(C)** Immediate post-ablation contrast-enhanced ultrasound showing no enhancement in the target area; **(D)** 1-month post-ablation panoramic ultrasound of ablation zone; **(E)** 1-month post-ablation contrast-enhanced ultrasound demonstrating no enhancement; **(F)** 1-month post-ablation enhanced MRI showing lesion size reduction compared to baseline; **(G)** 6-month post-ablation enhanced MRI indicating volume reduction in majority of lesions; **(H)** 6-month post-ablation ultrasound revealing no discernible mass echo in the region; **(I)** 1-year post-ablation enhanced MRI demonstrating significant regression of the lesion; **(J)** 2-year post-ablation enhanced CT showing no significant enhancement to suggest viable tumor; **(K)** 3-year post-ablation enhanced CT confirming persistent absence of enhancement; **(L)** 3-year post-ablation ultrasound with no detectable mass-like echo in the region.

Immediately, CEUS was performed post-MWA to evaluate the ablation zone, confirming complete tumor ablation (defined as absence of enhancement signals within the ablation area). A standardized follow-up protocol was implemented as follows:1 month, 3 months, 6 months and 12 months postoperatively (**Figure 2**): Combined assessment with CE-MRI and CEUS demonstrated progressive shrinkage of the ablation zone, with no pathological enhancement signals observed. Starting from the second postoperative year: Surveillance every 6 months included thoracic contrast-enhanced CT and breast CEUS, focusing on the original left breast lesion site and pulmonary disease control. Postoperative adjuvant therapy was tailored according to the National Comprehensive Cancer Network (NCCN) Clinical Practice Guidelines for Lung Adenocarcinoma, incorporating targeted therapy and involved-field radiotherapy. At the last follow-up (36 months postoperatively), imaging and clinical examinations revealed no local recurrence in the left breast or new metastatic lesions, with a progression-free survival (PFS) of 36 months.

## Discussion

Current standard therapeutic strategies for MBC primarily rely on systemic therapies (e.g., chemotherapy, targeted therapy) and local surgical resection (1, 7, 8, 10–13). However, the applicability of surgical intervention is constrained by patients’ general health status, metastatic lesion location, and multidisciplinary treatment goals (**Table 1**). Literature reports indicate that approximately 35-45% of MBC patients are ineligible for curative surgery due to distant metastases, poor performance status, or proximity of lesions to critical anatomical structures (14, 15). In this context, minimally invasive techniques such as radiofrequency ablation and MWA have emerged as viable alternative therapeutic options (16–19). MWA utilizes high-frequency electromagnetic waves to induce rapid oscillation of water molecules within tissues, generating thermal energy with unique advantages including high thermal efficiency (penetration depth: 3–5 cm), homogeneous ablation zones, and real-time monitoring capability (18, 20). Consequently, MWA demonstrates superior efficacy in overcoming the “heat sink effect” in fibrotic breast tissues, ensuring adequate ablation margins.

**Table 1:** Treatment of breast metastases from extramammary malignancies.

Category	Specific approach	Indications/Notes	References
General Principle	Systemic therapy is primary; local therapy is palliative	Breast metastasis indicates stage IV disease; treatment should target the primary tumor type and molecular profile.	Wang et al. (8); Vranic et al. (12); Longo et al. (9)
Systemic Therapy	Chemotherapy	Used for most solid tumors (e.g., lung, gastric, ovarian cancers).	Vaidya et al. (11) Makino et al. (10)
	Targeted Therapy	Based on driver mutations: EGFRmutations (NSCLC); ALK rearrangements; BRAF mutations (melanoma); HER2 (rare in metastases); BRCA mutations	Wang et al. (8) ol-31-04-15481 (13) Vranic et al. (12)
	Immunotherapy	PD-1/PD-L1 inhibitors	Makino et al. (10) Vranic et al. (12)
	Endocrine Therapy	For hormone receptor–positive metastases	Vranic et al. (12)
Local Therapy	Surgery	Reserved for: Symptomatic lesions; Solitary metastasis with controlled primary; Diagnostic confirmation Procedures:lumpectomy, simple mastectomy	Longo et al. (9); Makino et al. (10); Vaidya et al. (11)
	Radiotherapy	Palliative intent:Pain control;Local tumor control; Bone metastases with fracture risk	Longo et al. (9); Vaidya et al. (11)
Supportive Care	Pain management, bisphosphonates/denosumab for bone metastases, nutritional/psychological support	Integral part of care for all patients with advanced disease	Longo et al. (9)
Factors Influencing Treatment Choice	Primary tumor type Molecular biomarkers, Extent of metastatic spread, Patient performance status and comorbidities, rior treatment history and resistance patterns	Individualized treatment decisions require multidisciplinary review and often repeat biopsy for biomarker profiling.	Vranic et al. (12); Wang et al. (8); ol-31-04-15481 (13)

Notably, no prior studies have documented the application of MWA in MBC management. This case represents the first report of ultrasound-guided palliative MWA for lung adenocarcinoma-derived MBC. This case differs from primary early-stage malignancies in several key aspects: Firstly, the lesion’s large volume and proximity to both the skin and retromammary space necessitate meticulous protection of surrounding tissues to prevent thermal injury to the dermis, retro-mammary musculature, and lung parenchyma. Secondly, the tumor’s irregular morphology mandates expanded ablation margins to ensure complete coverage of the target area. Additionally, as metastatic disease requires multidisciplinary management, comprehensive treatment integrating complete tumor ablation with systemic therapies is essential.

Key technical innovations contributing to procedural success include: Preprocedural multimodal imaging integrating conventional ultrasound and CEUS precisely delineated tumor boundaries. A three-dimensional ablation pathway (superior-inferior, posterior-anterior, deep-superficial planes) was designed to ensure complete coverage of the lesion with a 10 mm safety margin. Peritumoral injection of a saline-lidocaine mixture (3:1 ratio) achieved dual objectives: thermal protection (preventing skin/chest wall injury) and enhanced antenna positioning accuracy through hydraulic tissue separation.Intraprocedural CEUS-guided intermittent assessment of non-enhancing zones ensured strict adherence to the endpoint of complete devascularization, achieving 100% immediate technical success.The patient subsequently received guideline-directed multimodal therapy, including targeted agents and cytotoxic chemotherapy.

Postoperative follow-up over 36 months demonstrated progressive volume reduction of the ablation zone with no local recurrence or new metastases, suggesting that MWA may disrupt the “metastasis-remetastasis” cascade via complete tumor cell inactivation. This observation aligns with mechanisms underlying long-term survival benefits observed in hepatocellular and pulmonary carcinoma ablation therapies.

## Conclusion

In conclusion, ultrasound-guided percutaneous MWA can be a safe and effective treatment for metastatic breast cancer patient and achieved durable local control.It may serve as a novel alternative approach for patients who underwent MBC.

## Data Availability

The original contributions presented in the study are included in the article/supplementary material. Further inquiries can be directed to the corresponding author.

## References

[1] Longo R Melgar E Campitiello M Plastino F Eid N Quirin I . Breast metastasis from squamous cell carcinoma of the oropharynx: a case report. J Med Case Rep. (2017) 11:355. doi: 10.1186/s13256-017-1500-3. PMID: 29268777 PMC5740581

[2] Semba R Horimoto Y Arakawa A Saito M . Metastatic breast tumors from extramammary Malignancies: a case series. Surg Case Rep. (2021) 7:154. doi: 10.1186/s40792-021-01235-2. PMID: 34185204 PMC8241943

[3] Dizdar O Aksoy S Altundag K . Is initially metastatic breast carcinoma different from recurrent metastatic breast carcinoma? Ann Oncol. (2009) 20:189. doi: 10.1093/annonc/mdn604. PMID: 32560007

[4] Bahloul N Ben Kridis W Yangui I Gargouri R Khmekhem R Boudawara T . Breast metastasis from lung cancer. Indian J Thorac Cardiovasc Surg. (2025) 41:1099–101. doi: 10.1007/s12055-025-01943-6. PMID: 40692991 PMC12276174

[5] Jeong YJ Bong JG Oh HK Park SH Kang SM Bae SH . Metachronous isolated breast metastasis from pulmonary adenocarcinoma with micropapillary component causing diagnostic challenges. BMC Cancer. (2014) 14:736. doi: 10.1186/1471-2407-14-736. PMID: 25274100 PMC4194376

[6] Mirrielees JA Kapur JH Szalkucki LM Harter JM Salkowski LR Strigel RM . Metastasis of primary lung carcinoma to the breast: a systematic review of the literature. J Surg Res. (2014) 188:419–31. doi: 10.1016/j.jss.2014.01.024. PMID: 24560348

[7] Mahindra SR Kinkhede D Pangarkar M Pagey R Deulkar S Devi Y . Metastasis to breast: a clinical and diagnostic challenge. Oncol J India. (2024) 8:48–51. doi: 10.4103/oji.oji_19_24

[8] Wang B Jiang Y Li SY Niu RL Blasberg JD Kaifi JT . Breast metastases from primary lung cancer: a retrospective case series on clinical, ultrasonographic, and immunohistochemical features. Transl Lung Cancer Res. (2021) 10:3226–35. doi: 10.21037/tlcr-21-542. PMID: 34430360 PMC8350075

[9] Longo R Melgar E Campitiello M Plastino F Eid N Quirin I . Breast metastasis from squamous cell carcinoma of the oropharynx: a case report. J Med Case Rep. (2017) 11:355. doi: 10.1186/s13256-017-1500-3. PMID: 29268777 PMC5740581

[10] Makino M Kusama H Hagiwara M Horimoto Y Sato E Ikeda N . A case of occult breast cancer diagnosed during immune checkpoint inhibitor treatment for recurrent metastatic lung cancer. Surg Case Rep. (2025) 11:n/a. doi: 10.70352/scrj.cr.25-0389. PMID: 41122452 PMC12536039

[11] Vaidya T Ramani S Rastogi A . A case series of metastases to the breast from extramammary Malignancies. Indian J Radiol Imaging. (2018) 28:470–5. doi: 10.4103/ijri.IJRI_218_18. PMID: 30662213 PMC6319108

[12] Vranic S Senarathne W Stafford P Poorman K Pockaj BA Gatalica Z . Biomarkers of targeted therapy and immuno-oncology in cancers metastatic to the breast. Appl Immunohistochem Mol Morphol. (2020) 28:661–8. doi: 10.1097/PAI.0000000000000808. PMID: 31517642 PMC7664953

[13] Yang T Xu Y Lin X Chen G Wang F . Metastatic breast cancer in primary lung cancer with compound EGFR mutations: a case report. Oncol Lett. (2026) 31:1–5. doi: 10.3892/ol.2026.15481. PMID: 41710084 PMC12910663

[14] Spasic M Zaric D Mitrovic M Milojevic S Nedovic N Sekulic M . Secondary breast Malignancy from renal cell carcinoma: challenges in diagnosis and treatment—case report. Diagnostics. (2023) 13:991. doi: 10.3390/diagnostics13050991. PMID: 36900135 PMC10000768

[15] Ko K Ro JY Hong EK Lee S . Micropapillary lung cancer with breast metastasis simulating primary breast cancer due to architectural distortion on images. Korean J Radiol. (2012) 13:249. doi: 10.3348/kjr.2012.13.2.249. PMID: 22438695 PMC3303911

[16] Burak WE Agnese DM Povoski SP Yanssens TL Bloom KJ Wakely PE . Radiofrequency ablation of invasive breast carcinoma followed by delayed surgical excision. Cancer. (2003) 98:1369–76. doi: 10.1002/cncr.11642. PMID: 14508822

[17] Dai Y Liang P Wang J Luo Y Yu X-L Han Z-Y . Microwave ablation without subsequent lumpectomy versus breast-conserving surgery for early breast cancer: a propensity score matching study. Int J Hyperthermia. (2023) 40:2186325. doi: 10.1080/02656736.2023.2186325. PMID: 36944374

[18] Pan H Qian M Chen H Wang H Yu M Zhang K . Precision breast-conserving surgery with microwave ablation guidance: a pilot single-center, prospective cohort study. Front Oncol. (2021) 11:680091. doi: 10.3389/fonc.2021.680091. PMID: 34123849 PMC8187871

[19] Izzo F Thomas R Delrio P Rinaldo M Vallone P DeChiara A . Radiofrequency ablation in patients with primary breast carcinoma: a pilot study in 26 patients. Cancer. (2001) 92:2036–44. doi: 10.1002/1097-0142(20011015)92:8<2036::AID-CNCR1542>3.0.CO;2-W 11596017

[20] Marcy P-Y Magné N Castadot P Bailet C Namer M . Ultrasound-guided percutaneous radiofrequency ablation in elderly breast cancer patients: preliminary institutional experience. Br J Radiol. (2007) 80:267–73. doi: 10.1259/bjr/91383984. PMID: 17068011

